# Withdrawing mycophenolate mofetil in treating a young kidney transplant recipient with COVID-19: A case report

**DOI:** 10.1097/MD.0000000000020481

**Published:** 2020-06-12

**Authors:** Dong Chen, Bo Yang, Yan Zhang, Liang Chen, Lai Wei, Weijie Zhang, Xinqiang Wang, Xiaolin Tong, Zhishui Chen

**Affiliations:** a Institute of Organ Transplantation, Tongji Hospital, Tongji Medical College, Huazhong University of Science and Technology, Wuhan; b Key Laboratory of Organ Transplantation, Ministry of Education, Ministry of Public Health, Chinese Academy of Medical Sciences, Wuhan; c General Medical Department, Tongji Hospital, Tongji Medical College, Huazhong University of Science and Technology, Wuhan; d Endocrinology Department, Guanganmen Hospital, Chinese Academy of Traditional Chinese Medicine, Beijing, China.

**Keywords:** COVID-19, immunosuppressant, kidney transplantation, renal function, traditional Chinese medicine

## Abstract

**Rationale:**

Coronavirus disease 2019 (COVID-19) is a novel infectious disease and became a global issue. Treatment of COVID-19 especially in solid organ transplant recipients is empirical and controversial, especially the adjustment of the immunosuppressants.

**Patient concerns:**

A 29-year-old kidney transplant recipient with the symptoms of COVID-19 pneumonia.

**Diagnoses:**

COVID-19 pneumonia after kidney transplantation.

**Interventions:**

He was treated with modified immunosuppressants (unchanged dose of tacrolimus and oral corticosteroids while discontinuing mycophenolate mofetil (MMF)), antibiotics, interferon α-2b inhalation and traditional Chinese medicine.

**Outcomes:**

He recovered from COVID-19 pneumonia after 29 days of hospitalization. And the renal function (measured as blood urea nitrogen, serum creatinine, and urine protein) returned to normal.

**Lessons:**

In certain group of COVID-19 (e.g., mild to moderate cases, young patients without comorbidities), a reduction instead of an overall withdrawal of immunosuppressant in kidney transplant recipients is feasible.

## 1 Introduction

The epidemic coronavirus disease 2019 (COVID-19) has spread worldwide rapidly.^[1,2]^ By March 28th, 2020, 571,678 patients from 6 regions were diagnosed and 26,494 died in this global pandemic.^[3]^

Kidney transplant recipients are vulnerable to infectious diseases because of chronic immunosuppression.^[4]^ Immunosuppressive therapy usually aggravates infectious status in kidney transplant recipients, including anti-lymphocyte therapy, calcineurin inhibitor (CNI, e.g., tacrolimus), high dose corticosteroids, and mycophenolate mofetil (MMF).^[5–8]^ Thus, individual therapy with a careful adjustment of immunosuppressive agents for kidney transplant recipients with COVID-19 is challenging.

Evidence of the pathological process, treatment, and prognosis of COVID-19 in kidney transplant recipients remains scarce. Questions regarding the efficiency in adjusting immunosuppressive drugs, the immune response to infection or rejection, and changes of renal function during the course of disease remain to be answered. Here, we present a young man of COVID-19 after kidney transplantation who was treated with unchanged dose of tacrolimus and oral corticosteroids while withdrawing MMF.

## 2 Case report

On January 20th, 2020, a 29-year-old man presented to the clinic with a 1-day fever (37.3°C) and dry cough. The patient lived in a local community of Wuhan city for years and did not have a travel history outside the city within 3 months before illness. He received a donated kidney from his father and underwent living-related kidney transplantation in July, 2010. Since then, immunosuppressive drugs were maintained, including tacrolimus, MMF, and methylprednisolone. Tacrolimus was administered 1 to 1.5 mg per 12 hours and the concentration remained 4.0 to 5.0 ng/mL. MMF was administered 500 mg every 12 hours and methylprednisolone 4 mg every day. The serum creatinine level of this patient was around 130 μmol/L. The daily urine volume was stable at about 2000 mL. Before this visit, he was generally fit and did regular follow-up at the outpatient department.

His latest follow-up recorded no physical discomfort on January 16th, 2020. Serum creatinine and urea level were 128 μmol/L and 9.30 mmol/L, respectively. Count of white blood cell and its classification remained in the normal rage. Urine protein was 2+ (Table 1).

**Table 1:**
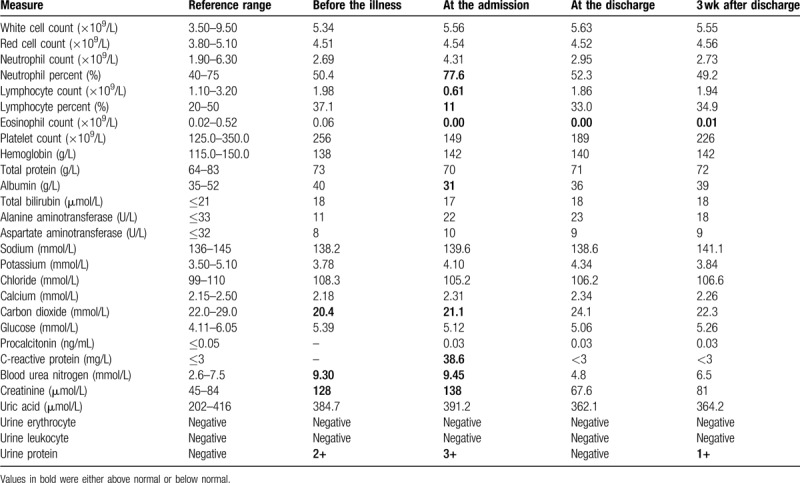
Clinical laboratory results.

**Figure 1:**
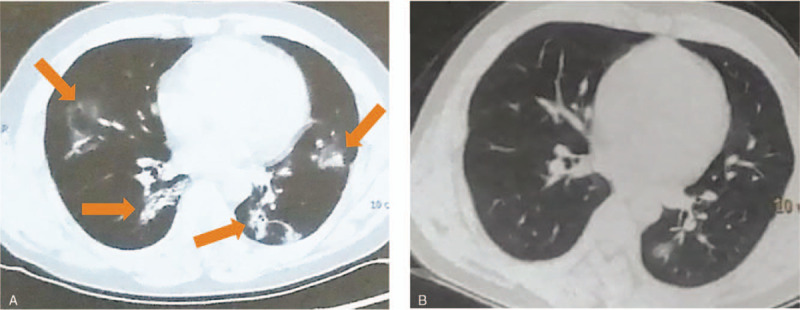
Chest CT images. (A) Exudative lesions were in the middle and lower lobe of the right lung and the lingular segment and lower lobe of the left lung. Middle lobe of the right lung and lingular segment of the left lung also showed ground glass opacity. Image was obtained on January 26th, 2020 (arrows). (B) After treatment, lesions in the middle and lower lobe of the right lung and lingular segment of the left lung were completely absorbed. Lesions in the lower lobe of the left lung were partially absorbed, manifested as a reduced lesion size, and density. Image was obtained on February 13th, 2020.

On January 20th, 2020, he was prescribed oral moxifloxacin (0.4, once a day) and oseltamivir (75 mg, twice a day) for 6 days because of fever. Fever persisted as a body temperature ranging from 37.6°C to 38.8°C together with frequent cough, chest tightness, shortness of breath, and finally breathlessness and dyspnea. On January 26th, his chest computed tomography (CT) scan showed multiple patchy ground glass opacity and exudative lesion in bilateral lungs (Fig. 1A). Blood oxygen saturation fluctuated from 80% to 90%. Count of lymphocyte dropped below the normal rage (Table 1). He was highly suspected of COVID-19 and admitted to the hospital. High flow oxygen therapy was applied. A nasopharyngeal swab specimen was obtained and sent for real-time reverse transcriptase polymerase chain reaction (RT-PCR) of severe acute respiratory syndrome coronavirus 2 (SARS-CoV-2). Result was positive and a diagnosis of COVID-19 was confirmed.

He was asked to stop taking MMF from January 26th, 2020. Tacrolimus and methylprednisolone were maintained at the previous dose. He received intravenous injection of Ceftazidime (2.0 g, twice a day), moxifloxacin (0.4 g, once a day), and interferon α-2b inhalation (5.0 million Units, twice a day). Meanwhile, he started to take traditional Chinese medicine decoction twice a day (radix paeoniae alba 30 g, lily 45 g, rhizoma atractylodis 45 g, roasted malt 30 g, pericarpium citri reticulatae 30 g, ligusticum chuanxiong hort 30 g, dangshen 30 g, licorice root 30 g, pogostemon cablin 30 g, officinal magnolia bark 30 g, forsythia 30 g, spina date seed 45 g, honey-fried licorice root 18 g, poria cocos 30 g, bran fried rhizoma atractylodis 30 g). The flow rate of oxygen therapy was adjusted according to the blood saturation of oxygen.

From February 5th, the patient felt better with body temperature decreased from 37.3°C to 36.5°C, blood oxygen saturation improved and remained around 95% when the oxygen therapy was temporarily removed for functional evaluation, and breathlessness and dyspnea disappeared. He still had some slight dry cough, and followed the above regimen for another 8 days. On February 13th, all the COVID-19 related symptoms of this patient disappeared. His second chest CT scan showed an obvious absorption of shadows in both lungs (Fig. 1B). The antibiotics and interferon α-2b were stopped, and therapeutic regimen remained only the traditional Chinese medicine. Later, 2 consecutive RT-PCR tests for SARS-CoV-2 was both negative. The patient was dismissed from the hospital on February 23rd. After that, he had routine examinations for white blood cells, red blood cells, platelet, renal function, and urine test. Counts of leukocytes and lymphocytes increased to normal level. Surprisingly, the serum creatinine and urea level returned to 67 μmol/L and 4.8 mmol/L, respectively and the urine protein was negative (Table 1).

Three weeks after discharge from the hospital, this patient had a regular examination recording no fever and other symptoms. The patient lost 5 kg of body weight during hospitalization. The serum creatinine and urea level remained 81 μmol/L and 6.5 mmol/L, respectively. The urine protein was 1+. Count of leukocyte and its classification were normal (Table 1). He was then allowed to take 250 mg MMF every 12 hours, and the dose of tacrolimus and methylprednisolone remained unchanged.

This study was approved by the institutional review board of Tongji Hospital, Tongji Medical College, Huazhong University of Science and Technology (TJ-IRB20200384). Written consent from this patient was obtained for the purpose of publication of the case details.

## 3 Discussions

The clinical symptoms, laboratory results, and chest CT findings of this case were similar to those of the general population with COVID-19.^[1]^ Although this young man developed symptoms of hypoxia, he finally recovered from oxygen therapy without mechanical ventilation. Like other infectious diseases after kidney transplantation (e.g., cytomegalovirus (CMV)), the intensity of immunosuppressive therapy can impact the treatment outcomes.^[9]^ Although complete discontinuation of immunosuppressant can promote the recovery of infection, it may cause allograft rejection and make the treatment more difficult. Up to now, 2 cases of COVID-19 in kidney transplant recipients were published.^[10,11]^ Standard immunosuppressants were withdrawn and only steroids were administered in both reports.^[10,11]^ In our case, however, only MMF was stopped until virus infection was controlled and the other 2 (methylprednisolone and tacrolimus) were continued all through the treatment. Outcomes turned out satisfying.

MMF inhibits the synthesis of guanine nucleotides and selectively prevents proliferation and activation of T and B lymphocytes.^[12]^ Tacrolimus can not only inhibit lymphocyte activation, but also effectively downregulate the infiltration of inflammatory cells, which might be helpful in the course of COVID-19.^[13]^ In addition, the adjuvant corticosteroid administration is being considered to modulate the immune response and counterbalance the systemic inflammatory reaction, which could also be helpful.^[14]^ Considering the downregulation of immune system without other possible benefit, the MMF was discontinued at the beginning and stepwise added after recovery while tacrolimus and corticosteroid were remained unchanged in this COVID-19 patient. Therefore, we suppose that under some certain circumstances (e.g., younger kidney transplant recipients without comorbidities, not needing mechanical ventilation) it is reasonable to selectively discontinue some types of immunosuppressant as practiced in other infections such as CMV and rejection episodes will not be unnecessarily provoked,^[9,15]^ even if interferon is given.

The use of glucocorticoids in the treatment of COVID-19 is also controversial. Some considered it helpful at appropriate dose,^[11,16]^ some argued for the scarce evidence of benefit, even in severe cases.^[17]^ In the present case, this patient did not receive intravenous corticosteroids. Only the oral methylprednisolone was maintained at the original dose after kidney transplantation. Interferon α-2b inhalation was prescribed to enhance the antiviral ability of the respiratory system albeit the risk of rejection.^[18]^ Again, a sustained immunosuppressant might help against allograft rejection after interferon α-2b inhalation. Meantime, Ceftazidime and moxifloxacin were used to prevent secondary infection. We also tried traditional Chinese herbs in this treatment therapy. The traditional Chinese herbs were reported to accelerate lung infiltrate absorption, decrease dosage of corticosteroids, and improve the quality of life in another highly pathogenic coronaviruses (i.e., severe acute respiratory syndrome).^[19]^ This patient recovered well from COVID-19 without allograft rejection and deterioration of renal function.

We were surprised that serum creatinine and urea level declined and urine protein turned negative after treatment of COVID-19. Three weeks after discharge, the serum creatinine, urea, and urine protein level still remained lower than before. We came up with 3 possible explanations. Firstly, reduced protein intake resulting from a loss of appetite during hospitalization could lead to a decreased generation of creatinine, urea, and urine protein.^[20–22]^ Secondly, regulatory T cell might play a role. According to the previous research, CD4 + T cells were induced and experienced an increase during the recovery phase of COVID-19.^[23]^ Regulatory T cells belong to CD4 + T cells, and was essential for alleviating inflammation and improving renal function in kidney transplant recipients.^[24,25]^ A hypothetical increase of regulatory T cell during the recovery period could act actively on renal function. However, this is mere a hypothesis and needed to be confirmed by solid evidence. Third, the traditional Chinese medicine may also play a role. Some traditional Chinese medicine prescribed in this case has been confirmed to protect kidney from immune injury and improve renal function, such as ligusticum chuanxiong hort.^[26–28]^ The limitation of the study was a short follow-up. Further outpatient monitoring should be continued for the short-term and long-term impact of COVID-19 on renal function of this renal transplant recipient.

Here we report 1 interesting case of COVID-19 infection after kidney transplantation who was treated with MMF withdrawing, unchanged dose of tacrolimus and oral corticosteroids, antibiotics, interferon α-2b inhalation, and traditional Chinese medicine. In certain group of COVID-19 (e.g., mild to moderate cases, young patients without comorbidities), a reduction instead of an overall withdrawal of immunosuppressant is feasible. However, this is a single case report and the outcomes cannot be extrapolated as treatment recommendation for transplant patient, and that studies about managing the immunosuppressive therapy when treating COVID-19 should be explored.

## Author contributions

**Conceptualization:** Zhishui Chen.

**Data curation:** Dong Chen.

**Formal analysis:** Bo Yang, Yan Zhang, Liang Chen.

**Funding acquisition:** Xiaolin Tong, Zhishui Chen.

**Investigation:** Lai Wei, Weijie Zhang.

**Methodology:** Yan Zhang, Liang Chen, Xinqiang Wang.

**Project administration:** Lai Wei, Weijie Zhang, Zhishui Chen.

**Resources:** Dong Chen.

**Supervision:** Zhishui Chen.

**Validation:** Zhishui Chen.

**Visualization:** Zhishui Chen.

**Writing – original draft:** Dong Chen, Bo Yang.

**Writing – review & editing:** Dong Chen, Bo Yang, Zhishui Chen.
